# Perioperative Syndecan-1 Dynamics During Cardiac Surgery: Associations with Operative Factors and Patient Characteristics

**DOI:** 10.3390/medicina62071305

**Published:** 2026-07-06

**Authors:** Tadas Cesnaitis, Tadas Lenkutis, Renata Paukstaitiene, Rasa Bukauskiene, Judita Andrejaitiene, Astra Vitkauskiene, Rimantas Benetis

**Affiliations:** 1 Heart Centre, Lithuanian University of Health Sciences, LT-44307 Kaunas, Lithuania; 2 Department of Anaesthesiology, Lithuanian University of Health Sciences, LT-44307 Kaunas, Lithuania; 3 Health Data Scientific Analytics Centre, Lithuanian University of Health Sciences, LT-44307 Kaunas, Lithuania; 4 Institute of Cardiology, Lithuanian University of Health Sciences, LT-44307 Kaunas, Lithuania; 5 Department of Laboratory Medicine, Lithuanian University of Health Sciences, LT-44307 Kaunas, Lithuania

**Keywords:** Syndecan-1, endothelial glycocalyx, cardiac surgery, coronary artery bypass grafting, cardiopulmonary bypass, aortic cross-clamping, ischemia–reperfusion injury

## Abstract

*Background and objectives:* Cardiac surgery with cardiopulmonary bypass (CPB) is associated with endothelial glycocalyx injury and perioperative endothelial dysfunction. Syndecan-1 is commonly used as a biomarker of glycocalyx shedding, but data on its perioperative changes and their relationship with operative and patient-related factors remain limited. The aim of this study was to evaluate perioperative Syndecan-1 dynamics during coronary artery bypass grafting (CABG) with CPB and to assess associations with ischemia–reperfusion exposure and patient characteristics. *Materials and methods:* This prospective observational study included 147 patients undergoing elective CABG with CPB. Syndecan-1 concentrations were measured at five time points: before induction of anaesthesia, immediately after aortic cross-clamp application, immediately after aortic declamping, on arrival at the ICU and 24 h after surgery. Perioperative changes were analysed using non-parametric tests, Spearman’s rank correlation analysis and mixed-effects modelling. The study was registered at ClinicalTrials.gov (NCT03491163; registered on 29 March 2018). *Results:* Syndecan-1 concentrations changed significantly over time (*p* < 0.001), increasing from a baseline median of 49.74 ng/mL to a peak of 147.78 ng/mL at ICU admission, followed by a partial decline to 65.26 ng/mL at 24 h. Aortic cross-clamp duration was weakly but significantly associated with Syndecan-1 concentration at ICU admission (r_s_ = 0.243, *p* = 0.003) and with perioperative increases from baseline to ICU admission (ΔS4-1: r_s_ = 0.196, *p* = 0.017) and from aortic clamping to ICU admission (ΔS4-2: r_s_ = 0.207, *p* = 0.012). No significant associations were observed between CPB duration and Syndecan-1 concentrations in univariable analyses. In the mixed-effects model, a significant non-linear temporal pattern of Syndecan-1 concentrations was observed (both linear and quadratic time terms, *p* < 0.001). Male sex (β = 0.247, *p* = 0.009) and aortic cross-clamp duration (β = 0.016, *p* = 0.005) were independently associated with higher Syndecan-1 concentrations, whereas smoking status, age, BMI, diabetes status, EuroSCORE II, and CPB duration were not independently associated. *Conclusions:* Syndecan-1 concentrations increase significantly during cardiac surgery with cardiopulmonary bypass, peaking at ICU admission and partially declining within 24 h. Aortic cross-clamping duration, but not total CPB duration, showed weak associations with glycocalyx shedding. Male sex was independently associated with higher Syndecan-1 concentrations. These findings support ischemia–reperfusion injury as an important contributor to endothelial glycocalyx shedding during cardiac surgery.

## 1. Introduction

The endothelial glycocalyx (EG) is a thin layer of proteoglycans (syndecans), glycosaminoglycans (heparan sulphate, hyaluronan), glycoproteins, and bound plasma proteins covering the luminal surface of blood vessels that regulates vascular permeability, mechanotransduction (shear stress sensing), leukocyte adhesion inhibition, anticoagulation, and microvascular homeostasis [1,2]. In addition to these functions, increasing evidence suggests that the endothelial glycocalyx contributes to molecular transport, blood pressure regulation and binding of key molecules involved in vascular homeostasis [3,4].

One important characteristic of EG is its ability to change its thickness and regenerate depending on damaging and restorative factors [5]. Systemic inflammation, fluid overload, hyperglycaemia, and trauma contribute to endothelial glycocalyx degradation [6,7,8]. This EG damage is followed by increased vascular permeability, increased leucocytes and platelet adhesion, which promotes atherosclerosis and thrombosis [9,10].

EG is constantly changing and it exists in a dynamic equilibrium between shedding and regeneration. This balance keeps it functioning properly [11,12].

Identifying factors associated with endothelial glycocalyx shedding and regeneration may help to develop strategies aimed at preserving glycocalyx integrity and to improve clinical outcomes. Plasma concentration of Syndecan-1 (SDC-1) is currently one of the best-characterised and most widely used biomarkers of glycocalyx injury, with multiple studies demonstrating a strong association between glycocalyx injury and elevated circulating levels of this proteoglycan [13,14,15].

Endothelial glycocalyx injury in cardiac surgery.

Cardiac surgery induces EG damage through multiple pathways:

- Ischemia–reperfusion injury: CPB causes global or regional ischemia followed by reperfusion. This triggers oxidative stress and enzymatic degradation of the glycocalyx [15,16,17,18].
- Systemic inflammatory response: Surgical trauma activates cytokines (e.g., IL-18), complement pathways, mast cell degranulation (releasing heparinase), matrix metalloproteinases—all contributing to EG shedding [15,19,20].
- Hypervolemia/hypovolemia as mechanical factors: Shear stress changes during CPB or volume loading can induce atrial natriuretic peptide release that promotes glycocalyx shedding [21].

Syndecan-1 as a Marker of Glycocalyx Shedding:

- SDC-1 and heparan sulphate are established plasma biomarkers for EG degradation and may help clarify how pathological processes influence EG integrity and help clinicians to evaluate future outcomes of the patient. SDC-1 concentration increases significantly during CPB or reperfusion phases [18,22,23].
- Elevated SDC-1 correlates with postoperative organ dysfunction, especially acute kidney injury (AKI), and may predict adverse outcomes in both adults and children undergoing cardiac surgery [24,25,26].

EG disruption leads to increased vascular permeability (oedema), prolonged postoperative impairment of microcirculatory perfusion [23], heightened risk for AKI [24,25,26], myocardial dysfunction [22], postoperative delirium [27], longer ICU/hospital stays [26], bleeding complications [28] and potentially higher mortality rates in severe cases.

Although endothelial glycocalyx injury during cardiac surgery has been increasingly investigated, the perioperative temporal profile of Syndecan-1 release and its relationship with specific operative factors remain insufficiently characterised. In particular, it remains unclear whether glycocalyx shedding is more closely associated with CPB duration itself or with ischemia–reperfusion processes related to aortic cross-clamping.

The primary hypothesis of this study was that greater ischemia–reperfusion exposure during cardiac surgery would be associated with increased endothelial glycocalyx shedding, reflected by higher perioperative Syndecan-1 concentrations. A secondary objective was to evaluate whether patient-related characteristics influence the magnitude of this response.

## 2. Materials and Methods

### 2.1. Study Design and Population

This prospective observational study was conducted at the Hospital of Lithuanian University of Health Sciences, Kaunas Clinics and approved by the Kaunas Regional Bio-medical Research Ethics Committee (Approval No. BE-2-1, 15 February 2017). The study was registered at ClinicalTrials.gov (Identifier: NCT03491163, registered on 29 March 2018). All patients aged 18–85 years who were scheduled for elective CABG with CPB and who met the inclusion criteria were informed about the possibility of participating in the trial during a one-year recruitment period. The exclusion criteria were: patients with low ejection fraction, previous cardiac surgery, rapid deterioration of the patient’s clinical condition, haematological, liver, renal disease, infection, use of glucocorticoids or other immunosuppressant treatment.

### 2.2. Anaesthesia

All patients received a peripheral intravenous and arterial line. Standard monitoring was performed before induction. Anaesthesia was induced using fentanyl, propofol, and rocuronium at weight-adjusted doses. After induction, a central venous catheter was placed in the internal jugular vein. Anaesthesia was maintained with sevoflurane (MAC 1.0–1.3%), fentanyl 10–12 µg/kg was used for analgesia. Total intraoperative crystalloid administration was restricted to 1000 mL.

### 2.3. Surgery and CPB

Coronary artery bypass grafting surgery was performed via median sternotomy. CPB was standardised across all cases, using a roller pump Stockert III and a Compactflo D703 oxygenator in full heparinization according to hospital protocol. The CPB circuit was primed with 1500 mL of Ringer’s acetate, supplemented with 10,000 IU of systemic heparin. Pump flow was maintained at a target cardiac index of 2.4 L/min/m^2^. Myocardial protection was achieved using cold hyperkalaemic St. Thomas cardioplegia, administered by the anaesthetist. Normothermia was maintained throughout the procedure with mean arterial pressure 60–70mmHg.

### 2.4. Syndecan-1 Biomarker

SDC-1 concentration was measured at five time points: before induction of anaesthesia (S1), immediately after aortic cross-clamp application (S2), immediately after aortic declamping (S3), on arrival at the ICU (S4) and 24 h after surgery (S5). Samples were transported to the hospital laboratory, where the blood serum was extracted and frozen. After a sufficient number of samples had been collected, SDC-1 was measured using the BioVendor—Laboratorní medicína a.s. HUMAN SYNDECAN-1 (CD138) solid phase sandwich ELISA kit. Measurements were made according to manufacturer’s recommendations. SDC-1 concentrations were obtained and expressed in ng/mL.

After obtaining SDC-1 data ten new variables were created to evaluate change between different measurement time points and were called Deltas (ΔS) (e.g., ΔS5-1, showing change between the first measurement—S1, and the 24 h postoperative measurement S5).

### 2.5. Additional Data

Demographic data of patients were gathered. Aortic cross-clamping time, CPB duration, and total surgery time, ICU and total hospital days were recorded.

### 2.6. Statistical Analysis

For data analysis we used SPSS 31 (IBM Corporation, Armonk, New York, NY, USA). The normality of quantitative data was assessed with Shapiro–Wilk test. Normally distributed quantitative data are presented as mean (standard deviation—SD), whereas non-normally distributed data are presented as median (minimum-maximum values) and interquartile range (IQR).

Quantitative variables that did not meet the normality assumption were analysed using the Mann—Whitney U test (for two independent groups) or the Kruskal–Wallis test (for more than two independent groups). Related quantitative variables were compared using the Friedman test.

Spearman’s rank correlation coefficient (rS) was used to estimate associations between SDC-1 concentrations and clinical or operative variables. *p*-values < 0.05 were considered statistically significant.

Longitudinal changes in SDC-1 concentrations were analysed using mixed-effects models to appropriately account for repeated measurements within individuals. Because SDC-1 values were right-skewed, concentrations were log-transformed prior to analysis. To account for the non-linear perioperative trajectory of SDC-1 concentrations, both linear and quadratic time terms (Time and Time^2^) were included in the model. Sex, smoking status, age, diabetes status, EuroSCORE II, CPB, aortic cross-clamp duration and body mass index (BMI) were included as fixed effects. A random intercept was specified for each individual (V1) to account for between-subject variability in baseline SDC-1 concentrations. Within-subject correlation across repeated measurements was modelled using a first-order autoregressive covariance structure [AR(1)].

## 3. Results

### 3.1. Patient and Surgery Characteristics

**Figure 1:**
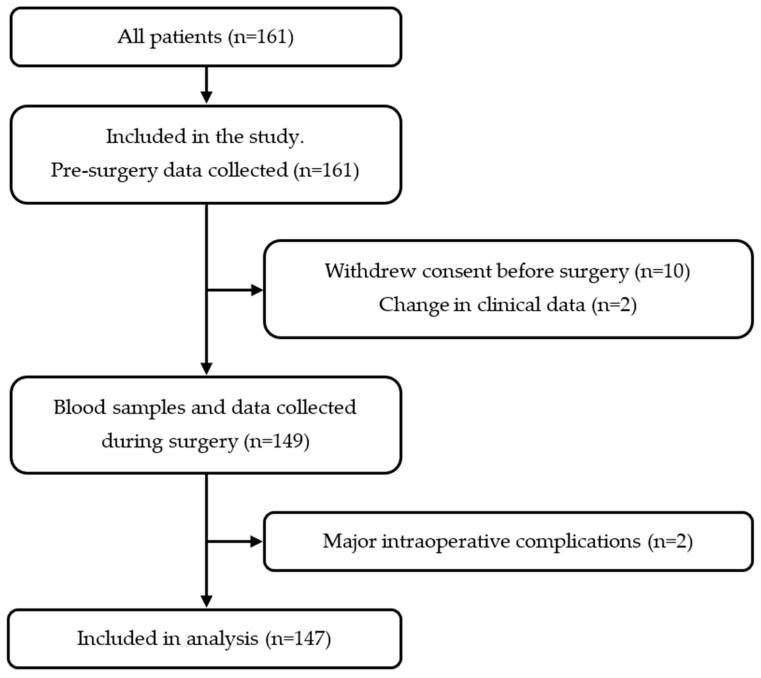
Patient flow chart of the study.

A total of 161 patients were selected for the study and complete datasets from 147 patients were included in the final analysis (Figure 1). Two patients who experienced major intraoperative complications resulting in death were excluded because complete perioperative biomarker measurements could not be obtained.

Patient characteristics are presented in Table 1. Surgical characteristics are presented in Table 2.

### 3.2. Perioperative Syndecan-1 Dynamics

**Figure 2:**
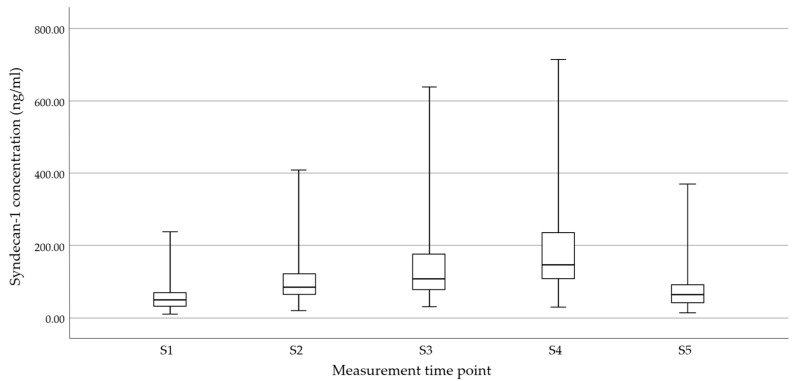
Perioperative Syndecan-1 concentrations during cardiac surgery. Boxplots represent median values and interquartile ranges; whiskers represent non-outlier ranges.

Our study found statistically significant changes in SDC-1 concentration during cardiac surgery. A Friedman test demonstrated a significant difference in SDC-1 concentrations across the five time points (*p* < 0.001). Pairwise comparisons revealed statistically significant differences in concentrations between all measurement time points (for all comparisons *p* < 0.001). Median values increased progressively from S1 to S4 and decreased at S5 (Table 3), (Figure 2).

SDC-1 concentration changes were calculated between different measurement times (Table 4).

### 3.3. CPB, Aortic Cross-Clamping and Surgery Time

**Figure 3:**
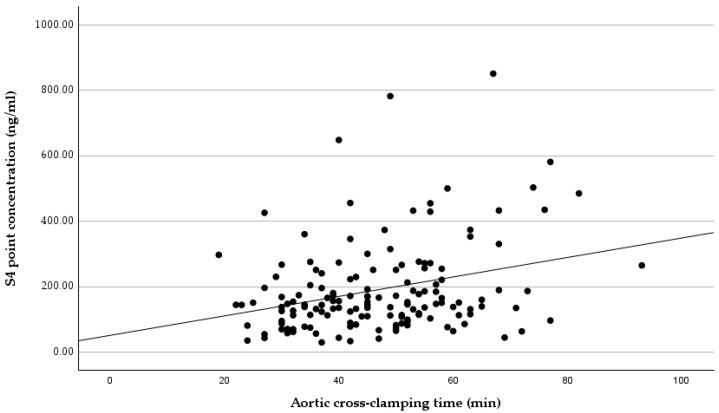
Scatterplot of S4 time point Syndecan-1 concentration correlation with aortic cross-clamping time.

Statistical analysis showed that there was no statistically significant relation between SDC-1 concentration or its changes and CPB time. However, a modest statistically significant correlation was observed between SDC-1 concentration and aortic cross-clamping time. SDC-1 concentration correlated significantly with aortic cross-clamping time at the S4 time point (rS = 0.243, *p* = 0.003; 95% CI: 0.080–0.394) (Table 5), (Figure 3).

Also, data analysis showed that biomarker elevation from S1 to S4 (ΔS4-1) (rS = 0.196, *p* = 0.017; 95% CI: 0.030–0.351) and from S2 to S4 (rS = 0.207, *p* = 0.012; 95% CI: 0.042–0.361) were weakly but statistically significantly related to aortic cross-clamping time. In contrast, later changes between ICU admission and 24 h (ΔS5-4) were inversely associated with both aortic cross-clamping and total surgery time (rS = −0.21, *p* = 0.01) (Table 6).

### 3.4. Patient Sex, Age, BMI, Smoking Status and SDC-1 Dynamics

Age showed weak correlations with perioperative Syndecan-1 dynamics. Significant negative correlations were observed between age and ΔS3-1 (r_s_ = −0.188, *p* < 0.05) as well as ΔS3-2 (r_s_ = −0.205, *p* < 0.05), indicating smaller early perioperative increases in older patients. In contrast, age demonstrated a weak positive correlation with ΔS5-3 (r_s_ = 0.250, *p* < 0.05). No significant associations were identified between age and absolute Syndecan-1 concentrations at individual time points.

BMI demonstrated weak positive correlations with S4 Syndecan-1 concentrations (r_s_ = 0.180, *p* < 0.05), ΔS2-1 (r_s_ = 0.187, *p* < 0.05), and ΔS4-1 (r_s_ = 0.200, *p* < 0.05). A weak negative correlation was observed between BMI and ΔS5-4 (r_s_ = −0.232, *p* < 0.05). No other significant associations between BMI and Syndecan-1 concentrations or delta changes were identified.

Male patients demonstrated significantly higher perioperative SDC-1 concentrations at S3 compared with female patients, 134.58 vs. 90.92 ng/mL (Z = −3.12, *p* = 0.002). Males also showed greater perioperative increases in SDC-1, including ΔS3–1 (67.02 vs. 37.71 ng/mL, Z = −3.07, *p* = 0.002) and ΔS3-2 (35.48 vs. 13.11 ng/mL, Z = −3.31, *p* < 0.001), suggesting a more pronounced early endothelial glycocalyx shedding response during surgery. In contrast, females demonstrated smaller postoperative declines, reflected by less negative ΔS5-3 values (-23.74 vs. −58.61 ng/mL, Z = −3.93, *p* < 0.001), and slightly higher residual SDC-1 elevation at 24 h, shown by ΔS5-1 differences (19.83 vs. 7.11 ng/mL, Z = −2.03, *p* = 0.042). No significant sex-related differences were observed at baseline, S2, S4, or most other delta measurements.

Smoking status was associated with lower late perioperative SDC-1 concentrations. Smokers had significantly lower S4 values at ICU admission compared with non-smokers (128.89 vs. 165.40 ng/mL, Z = −2.62, *p* = 0.009). Non-smokers also demonstrated greater perioperative increases in SDC-1, including ΔS4-1 (105.74 vs. 80.80 ng/mL, Z = −2.40, *p* = 0.017), ΔS4-2 (75.94 vs. 41.17 ng/mL, Z = −2.09, *p* = 0.037), and ΔS4-3 (47.42 vs. 6.94 ng/mL, Z = −3.46, *p* < 0.001). Smokers showed a steeper decline after peak SDC-1 concentrations, reflected by more negative ΔS5-3 values (−63.34 vs. −41.04 ng/mL, Z = −2.29, *p* = 0.022).

To further evaluate the independent effects of patient and perioperative factors on longitudinal SDC-1 concentrations, a mixed-effects model adjusted for demographic, clinical, and operative variables was constructed.

Mixed-effects modelling (Table 7) demonstrated a significant non-linear temporal pattern of SDC-1 concentrations throughout the perioperative period. Both the linear time term (β = 1.394, *p* < 0.001) and the quadratic time term (β = −0.213, *p* < 0.001) were significant, indicating progressive increases in SDC-1 concentrations during surgery followed by a decline at 24 h postoperatively.

Sex remained independently associated with SDC-1 concentrations. Male patients demonstrated significantly higher SDC-1 concentrations compared with females (β = 0.247, *p* = 0.009), corresponding to approximately 28.1% higher average SDC-1 levels (95% CI 6.4–54.2%).

Among perioperative factors, aortic cross-clamp duration was independently associated with SDC-1 concentrations (β = 0.016, *p* = 0.005). Each additional minute of aortic cross-clamping was associated with an estimated 1.6% higher SDC-1 concentration (95% CI 0.5–2.6%) after adjustment for patient characteristics and other perioperative variables. In contrast, cardiopulmonary bypass duration was not independently associated with SDC-1 concentrations.

Although smokers demonstrated significantly lower SDC-1 concentrations at selected perioperative time points in subgroup analyses, smoking status was not independently associated with overall SDC-1 concentrations in the mixed-effects model (β = −0.150, *p* = 0.134).

Age, BMI, diabetes status and EuroSCORE II were not independently associated with SDC-1 levels. Nevertheless, these covariates were included to control for potential confounding.

The AR(1) correlation parameter was statistically significant (r = 0.156; *p* = 0.025), confirming a correlation between repeated measurements within individuals. The random intercept variance was also significant, highlighting meaningful between-subject variability in baseline SDC-1 concentrations.

## 4. Discussion

This study demonstrates significant perioperative alterations in SDC-1 concentrations during cardiac surgery with CPB, reflecting dynamic endothelial glycocalyx disruption and suggesting that selected demographic characteristics may influence this response. Multiple intraoperative factors during cardiac surgery may contribute to endothelial glycocalyx degradation, as described by Knezevic et al. [29]. Given the multifactorial nature of this process, the present study focused on systemic endothelial glycocalyx injury rather than isolated myocardial endothelial damage, unlike Passov et al., who assessed coronary sinus blood during the early reperfusion phase [16].

The data demonstrated a progressive increase in SDC-1 from baseline to a peak at ICU admission (S4), followed by partial recovery at 24 h (S5) (*p* < 0.001). The peak at S4 suggests that maximal injury occurs late intraoperatively or immediately postoperatively, rather than during a single isolated phase. The subsequent decline at S5, despite not returning to baseline levels, indicates that endothelial glycocalyx injury is a dynamic process, with progressive intraoperative damage followed by partial recovery during the first 24 postoperative hours.

A weak but statistically significant association was found between aortic cross-clamping duration and SDC-1 levels, particularly at S4 (r_s_ = 0.243, *p* = 0.003) and with perioperative increases such as ΔS4-1 and ΔS4-2. Although these findings suggest that longer ischemic periods may contribute to endothelial glycocalyx shedding, the observed effect sizes were modest, indicating that aortic cross-clamp duration alone explains only a limited proportion of the variability in SDC-1 concentrations. Global ischemia and altered blood flow parameters are recognised mechanisms that may disrupt endothelial glycocalyx integrity. The progressive increase in SDC-1 concentrations from S2 (immediately after cross-clamp application) to S4 (ICU admission) is consistent with a potential contribution of ischemia–reperfusion injury to glycocalyx degradation [15,30], although causality cannot be established from the present data and other perioperative factors are likely involved. Importantly, aortic cross-clamp duration remained independently associated with SDC-1 concentrations in the adjusted mixed-effects model, suggesting that ischemic exposure contributes to glycocalyx shedding even after accounting for patient characteristics and other perioperative variables. The inverse association observed for ΔS5–4 (r_s_ = −0.212, *p* = 0.01) further suggests that prolonged aortic cross-clamping may be linked to a more pronounced post-peak decline in SDC-1. This pattern could reflect greater early glycocalyx shedding during ischemia–reperfusion, followed by subsequent clearance or redistribution of circulating SDC-1; however, these mechanisms were not directly assessed in the present study.

This study did not demonstrate a statistically significant independent association between CPB duration and SDC-1 concentrations. The literature contains mixed findings regarding CPB and SDC-1 associations. Dekker et al. and Robich et al. reported that longer CPB duration was associated with higher SDC-1 concentrations [23,31]. In contrast, Bol et al. reported no correlation [32]. These discrepancies may be partly explained by differences in study size, operative techniques, and perioperative management. Collectively, the available evidence suggests that CPB duration alone is unlikely to fully explain the extent of endothelial glycocalyx injury during cardiac surgery. Additionally, glycocalyx injury may depend on multiple intraoperative factors—such as perfusion and surgery strategy, shear stress exposure, temperature management, and circuit parameters.

The observed SDC-1 changes and their association with aortic cross-clamp duration support the hypothesis that ischemia–reperfusion contributes to endothelial glycocalyx injury during cardiac surgery. Given the reported associations between glycocalyx degradation and postoperative organ dysfunction in previous studies, identification of perioperative factors associated with glycocalyx shedding may help guide future endothelial-protective strategies. Although the detected correlations were modest, their consistency and biological plausibility support the utility of SDC-1 as a marker of intraoperative endothelial stress.

Regarding patient-related factors, study findings suggest that sex may be an important determinant of endothelial glycocalyx shedding during cardiac surgery. Male patients demonstrated approximately 28.1% higher SDC-1 concentrations than females in the adjusted mixed-effects model. Previous studies have reported higher circulating Syndecan-1 concentrations in males and have suggested sex-related differences in glycocalyx biology, potentially mediated by hormonal regulation of glycocalyx maintenance and shedding [33,34]. However, the mechanisms underlying these differences remain incompletely understood and higher circulating SDC-1 concentrations do not necessarily indicate a thicker glycocalyx. Therefore, the observed association should be interpreted cautiously and requires further investigation.

The smoking-related findings were unexpected. Smokers demonstrated lower SDC-1 concentrations at ICU admission and smaller perioperative increases in SDC-1 compared with non-smokers. Given the well-established association between smoking and endothelial dysfunction, the mechanisms underlying this observation remain unclear. Possible explanation is that chronic smoking may induce long-term alterations in endothelial glycocalyx due to atherosclerotic process, or affect the release and clearance of circulating glycocalyx components. However, this remains speculative. Smoking status was not independently associated with overall SDC-1 concentrations in the mixed-effects model, suggesting that the observed differences may be phase-specific rather than reflecting a consistent effect throughout the perioperative period. Furthermore, the relatively small number of smokers and the exploratory nature of the subgroup analyses warrant cautious interpretation. Confirmation in larger cohorts is required before firm conclusions can be drawn.

Age, BMI, diabetes status, and EuroSCORE II were not independently associated with overall SDC-1 concentrations in the adjusted mixed-effects model, although weak associations with selected perioperative SDC-1 changes were observed in univariable analyses. Similar observations have been reported previously [35].

These findings may also have implications for perioperative endothelial protection during cardiac surgery. Increasing evidence indicates that preservation of endothelial glycocalyx integrity may contribute to reducing microvascular dysfunction, inflammatory activation, and postoperative organ injury during cardiopulmonary bypass-supported procedures [18,19,36,37]. Glycocalyx degradation during cardiac surgery has been associated with ischemia–reperfusion injury, oxidative stress, inflammatory cytokine release, haemodilution, and altered perfusion conditions during extracorporeal circulation [18,19,36,37].

In this context, several perioperative strategies have been proposed to reduce endothelial injury and glycocalyx shedding. These include minimising ischemic exposure, avoiding unnecessarily prolonged aortic cross-clamping, and optimising perfusion during CPB [35]. Individualised perfusion management aimed at maintaining adequate oxygen delivery and stable hemodynamic conditions may help attenuate endothelial stress during surgery [36]. In addition, restrictive and balanced fluid administration, avoidance of excessive crystalloid-induced haemodilution, and modulation of perioperative inflammatory responses have been discussed as potential glycocalyx-preserving approaches [19,31,37].

Although the clinical benefit of these strategies has not yet been fully established, current evidence suggests that endothelial glycocalyx injury may represent a modifiable component of perioperative pathophysiology rather than an unavoidable consequence of CPB. Further studies combining glycocalyx biomarkers with clinical outcomes and microcirculatory assessment may help clarify the role of endothelial-protective perioperative strategies in cardiac surgical patients.

Several limitations should be considered. First, this was a single-centre observational study, which may limit generalizability of the findings. Because of the observational design, the identified associations cannot be interpreted as direct cause-and-effect relationships. Second, multiple correlation and subgroup analyses were performed, increasing the possibility of type I error. Third, perioperative haemodilution associated with cardiopulmonary bypass may have influenced measured SDC-1 concentrations and therefore affected the interpretation of absolute biomarker values. In addition, information regarding perioperative transfusion requirements was not available for the present analysis and therefore could not be evaluated. Furthermore, the relatively weak effect sizes indicate that additional, unmeasured factors contribute to glycocalyx shedding. Finally, the absence of direct measures of endothelial function or microcirculatory perfusion constrains mechanistic interpretation of the observed associations.

## 5. Conclusions

In this study Syndecan-1 concentrations increased significantly during cardiac surgery, peaking at ICU admission and declining within 24 h, indicating a substantial but partially reversible endothelial glycocalyx injury. Aortic cross-clamping duration was associated with greater glycocalyx shedding and remained an independent predictor of Syndecan-1 concentrations, supporting a contribution of ischemia–reperfusion-related endothelial injury.

Among patient-related factors, male sex was independently associated with higher SDC-1 concentrations, suggesting a more pronounced endothelial glycocalyx response during cardiac surgery. Smoking status, age, and body mass index, diabetes status and EuroSCORE II were not independently associated with overall SDC-1 concentrations, although selected subgroup analyses demonstrated weak phase-specific differences.

These findings support the use of Syndecan-1 as a marker of perioperative endothelial glycocalyx injury and highlight the potential importance of ischemic exposure during cardiac surgery. Further studies are required to determine whether perioperative glycocalyx shedding is associated with postoperative clinical outcomes and whether glycocalyx-protective strategies may improve patient outcomes.

## Figures and Tables

**Table 1:** Demographic data of the patients.

Characteristics	Overall (*n* = 147)
Age, years mean (SD)	66.6 (9.094)
Male sex, *n* (%)	93 (63.3)
Diabetic (Type-II), *n* (%)	39 (26.5)
Smokers, *n* (%)	40 (27.2)
EuroSCORE II, median (min–max) IQR	1.6 (0.6–13.6) 1.4
BMI median (min–max) IQR	29.26 (20.70–43.85) 5.74

**Table 2:** Surgery characteristics.

Characteristics	Overall (*n* = 147)
Number of anastomoses, *n* (%)	1–2 (1.4), 2–6 (4.1), 3–68 (46.3), 4–57 (38.3), 5–12 (8.2), 6–2 (1.4)
Aortic cross-clamping time (min) median (min–max) IQR	45 (19–93) 20
Bypass time (min) median (min–max) IQR	90 (46–163) 24
Surgery time (min) median (min–max) IQR	260 (185–390) 85
ICU stay (days) median (min–max) IQR	3 (2–21) 2
Hospital stay (days) median (min–max) IQR	11 (6–56) 4

**Table 3:** Syndecan-1 concentrations (ng/mL).

Time Point	Median (Min–Max)	IQR	Friedman χ^2^; *p*-Value
S1	49.74 (9.95–234.60)	39.15	343.68; *p* < 0.001
S2	85.74 (20.09–468.75)	57.62	
S3	108.65 (31.44–766.54)	99.61	
S4	147.78 (29.93–851.44)	132.95	
S5	65.26 (13.50–519.65)	50.27	

**Table 4:** Calculated changes (Δ) in Syndecan-1 concentrations (ng/mL).

Time Interval	Median (Min–Max)	IQR	Friedman χ^2^; *p*-Value
ΔS2-1	27.73 (−36.67–332.75)	43.31	766.38; *p* < 0.001
ΔS3-1	59.15 (−33.19–694.14)	91.46	
ΔS4-1	99.11 (−52.77–779.04)	120.92	
ΔS5-1	12.36 (−121.85–435.41)	38.47	
ΔS3-2	24.69 (−129.45–679.04)	68.37	
ΔS4-2	65.15 (−140.7–763.94)	99.29	
ΔS5-2	−21.53 (−333.95–425.57)	64.44	
ΔS4-3	40.49 (−442.72–384.64)	96.61	
ΔS5-3	−46.98 (−664.89–270.63)	83.41	
ΔS5-4	−80.67 (−749.79–222.45)	99.57	

**Table 5:** Correlations of time points and surgery event time.

	Time Point	Aortic Cross-Clamping Time	CPB Time	Surgery Time
Spearman’s rho (*p*-value)	S1	0.179 (0.030)	0.104 (0.211)	−0.050 (0.548)
	S2	0.138 (0.095)	0.099 (0.234)	−0.127 (0.126)
	S3	0.152 (0.067)	0.067 (0.422)	−0.017 (0.840)
	S4	0.243 (0.003)	0.134 (0.106)	−0.027 (0.742)
	S5	0.108 (0.191)	0.055 (0.509)	−0.044 (0.599)

Data presented as Spearman’s rho correlation (rS) with *p*-value.

**Table 6:** Correlations of Δ values and surgery event time.

	Δ Values	Aortic Cross-Clamping Time	CPB Time	Surgery Time
Spearman’s rho (*p*-value)	ΔS2-1	−0.004 (0.963)	0.043 (0.604)	−0.124 (0.136)
	ΔS3-1	0.051 (0.536)	0.034 (0.679)	0.029 (0.727)
	ΔS4-1	0.196 (0.017)	0.126 (0.127)	0.008 (0.92)
	ΔS5-1	−0.056 (0.503)	0.008 (0.925)	0.032 (0.697)
	ΔS3-2	0.098 (0.239)	0.056 (0.497)	0.147 (0.075)
	ΔS4-2	0.207 (0.012)	0.121 (0.143)	0.085 (0.305)
	ΔS5-2	−0.027 (0.749)	0.021 (0.805)	0.179 (0.03)
	ΔS4-3	0.205 (0.013)	0.148 (0.073)	0.007 (0.933)
	ΔS5-3	−0.1 (0.226)	−0.038 (0.649)	−0.1 (0.226)
	ΔS5-4	−0.212 (0.01)	−0.114 (0.168)	−0.212 (0.01)

Data presented as Spearman’s rho correlation (rS) with *p*-value.

**Table 7:** Mixed-effects model of longitudinal log-transformed Syndecan-1 concentrations adjusted for patient and perioperative factors.

Factors	β (Coefficient) Log-Scale	95% CI of β	*p*-Value	Exp (β)	95% CI of EXP (β)
Time (measurement order, S1–S5)	1.394	1.277–1.511	<0.001	4.031	3.585–4.533
Time^2^ (measurement order, S1–S5)	−0.213	−0.233–0.194	<0.001	0.808	0.792–0.823
Aortic cross-clamp time	0.016	0.005–0.026	0.005	1.016	1.005–1.026
CPB time	−0.008	−0.015–0.010	0.060	0.992	0.985–1.01
Sex (Male vs. Female)	0.247	0.062–0.433	0.009	1.281	1.064–1.542
Smoking (Yes vs. No)	−0.150	−0.347–0.047	0.134	0.860	0.707–1.048
Age	0.003	−0.007–0.013	0.385	1.003	0.993–1.013
BMI	0.002	−0.018–0.022	0.872	1.002	0.982–1.022
Diabetes	0.091	−0.108–0.290	0.368	1.095	0.898–1.336
EuroSCORE II	−0.017	−0.068–0.035	0.525	0.984	0.934–1.035

## Data Availability

The data presented in this study are available upon request from the corresponding author.

## Abbreviations

The following abbreviations are used in this manuscript:

SDC-1	Syndecan-1
EG	Endothelial glycocalyx
CABG	Coronary artery bypass grafting
CPB	Cardiopulmonary bypass
ICU	Intensive care unit
IQR	Interquartile range
AKI	Acute kidney injury
